# Durable, Anatomically Confined Clearance of Scalp Psoriasis and Associated Pruritus After Single Bilateral Greater Occipital Nerve Block

**DOI:** 10.7759/cureus.113086

**Published:** 2026-07-21

**Authors:** Jag Lally, Jessica L Forman, Margaret Puelle, Christopher Ritchlin, Francisco Tausk

**Affiliations:** 1 Dermatology, University of Rochester School of Medicine and Dentistry, Rochester, USA; 2 Dermatology, University of Rochester Medical Center, Rochester, USA; 3 Psychiatry, University of Rochester Medical Center, Rochester, USA; 4 Rheumatology, University of Rochester Medical Center, Rochester, USA

**Keywords:** greater occipital nerve block, lidocaine, lidocaine injection, pruritus, psoriasis treatment, scalp psoriasis

## Abstract

Psoriasis is a chronic, immune-mediated inflammatory skin disease that frequently involves the scalp, presenting as well-demarcated, erythematous plaques with silvery scale, pruritus, and impaired quality of life. Emerging evidence supports the role of sensory nerves in psoriatic inflammation through neuroimmune signaling involving the calcitonin gene-related peptide (CGRP) and interleukin-23/interleukin-17 (IL-23/IL-17) axis. Here, we report a case of severe, recalcitrant, markedly pruritic scalp psoriasis in a patient who failed multiple topical therapies and had only temporary improvement with apremilast before denial by her insurance, requiring discontinuation. Given the proposed neuroimmune pathophysiology of psoriasis, the patient underwent bilateral greater occipital nerve block utilizing lidocaine. At 169 days post-procedure, she demonstrated complete resolution of itch and clearance of plaques restricted to the anatomical territory of the greater occipital nerves, despite persistent disease in the adjacent scalp, without concomitant topical or systemic therapy. This sustained, anatomically confined response suggests that local nerve blockade represents a simple, inexpensive, in-office adjunct therapy for recalcitrant scalp psoriasis.

## Introduction

Psoriasis is an inflammatory skin disease that affects approximately 3% of adults in the United States and an estimated 125 million individuals worldwide [1,2]. The most common subtype, plaque psoriasis, is characterized by well-demarcated erythematous plaques with overlying silvery or micaceous scale, typically affecting the scalp, trunk, lumbosacral area, and extensor surfaces. Associated symptoms include pain, pruritus, scaling, bleeding, and impaired quality of life [1]. Diagnosis is often clinical, using morphologic features and distribution, with skin biopsy reserved for atypical presentations or diagnostic uncertainty [1]. The etiology of psoriasis is multifactorial, with both genetic variants and environmental triggers leading to dysregulated innate and adaptive immune signaling, specifically through the tumor necrosis factor-α (TNF-α), interleukin-23 (IL-23), and interleukin-17 (IL-17) pathways [1,3]. The pathogenesis of psoriasis has increasingly implicated a neuroimmune axis, in which sensory nerves have been shown to influence both pruritus and inflammation [4]. Central and peripheral denervation have been reported to improve psoriasis clinically, with experimental studies implicating a role for calcitonin gene-related peptide (CGRP) in sensory nerve signaling within the IL-23 and IL-17 pathway [5].

Given the association of psoriatic plaques with sensory nerve dysfunction, we hypothesized that scalp psoriasis may involve the greater occipital nerves and that blockage of these nerves may improve the disease. The greater occipital nerves are paired bilateral sensory nerves originating predominantly from the C2 dorsal rami, and provide cutaneous innervation to the posterior scalp extending upward toward the vertex of the scalp [6]. A nerve block is a therapeutic intervention in which a local anesthetic is injected near a peripheral nerve to transiently disrupt sensory signaling to the respective nerve’s target area [7]. Thus, a single, bilateral greater occipital nerve block refers to a single session in which lidocaine is injected around both greater occipital nerves. Reported adverse effects of occipital nerve blocks are typically mild, including injection-site pain, erythema, swelling, dizziness, vasovagal symptoms, and infection, though rarely patients have experienced nerve injury [7,8]. Although denervation injury and regional anesthesia have been associated with improvement in psoriasis, durable and anatomically confined clearance of scalp psoriasis after a single, office-based bilateral greater occipital block procedure has not been previously described, to our knowledge [5,9]. As such, we report a case of recalcitrant scalp psoriasis that achieved prolonged rash clearance and itch resolution confined to the distribution of the greater occipital nerves after a single bilateral lidocaine nerve block.

## Case presentation

A 66-year-old female was followed in the dermatology clinic for 3 years for severe scalp psoriasis, pruritus, and associated psoriatic alopecia. At the initial presentation in August 2023, she had well-demarcated salmon-colored plaques with silvery, micaceous scale involving the temporal, parietal, and occipital scalp, including significant postauricular involvement (Figure 1). The diagnosis of scalp psoriasis was made clinically, given the characteristic morphology. Over the following two years, the patient failed multiple topical therapies, including fluocinonide, coal tar shampoo, calcipotriene/betamethasone solution, ketoconazole shampoo, and roflumilast foam (Table 1).

**Figure 1:**
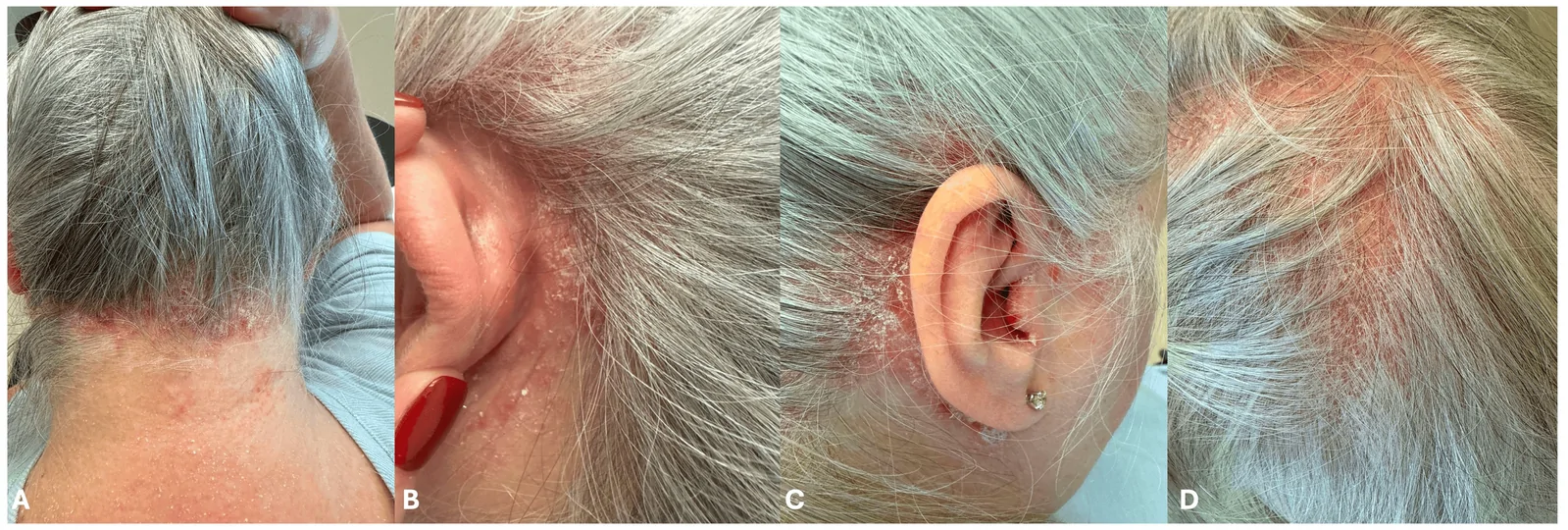
Scalp psoriasis prior to intervention A). Occipital scalp B). Left postauricular scalp C). Right postauricular scalp D). Vertex scalp

**Table 1:** Attempted therapies and clinical response, prior to nerve block PRN: as needed, OTC: over the counter

Therapy	Formulation and Dose	Frequency	Approximate Duration	Clinical Response
Fluocinonide solution	0.05% Oil	PRN	Single Application	Patient directed discontinuation after single use due to concern for hair shedding
Coal tar shampoo	OTC product	Every other day	Intermittent 2023-2025	Insufficient improvement
Calcipotriene/betamethasone dipropionate solution	0.005%/0.064%	Twice daily as needed	Intermittent	Worsened pruritus with compounded formulation
Ketoconazole shampoo	2%	2-3 times weekly (5-minute contact)	Intermittent 2023-2025	Insufficient improvement
Roflumilast foam	0.3%	Twice daily	January 2025 - present	Insufficient improvement
Apremilast (oral)	30 mg	Twice daily (after titration)	January 2025 - March 2025	Marked improvement; subsequent flare after insurance denial

In January 2025, the patient had marked improvement of rash and itch with 30 mg of apremilast twice daily; however, insurance subsequently denied coverage, which led to severe flaring of the scalp psoriasis. By September 2025, after re-trial of multiple topical therapies (Table 1), the patient continued to have uncontrolled disease with severe pruritus and associated hair loss, impairing her quality of life. At this visit, the patient underwent a bilateral greater occipital nerve block with a total of 6 mL of lidocaine 1% (3 mL per side). At a follow-up 20 days later, the patient reported significant improvement in itch and was not using topical medications. She denied adverse effects from the procedure.

In February 2026, the patient presented for a follow-up appointment, more than 5 months (169 days) after the nerve block. At that time, the patient noted persistent clearance of psoriasis and resolution of itch throughout the frontal, parietal, and vertex scalp. Notably, the patient had not used any systemic or topical therapy since the September 2025 visit. Moderate levels of pruritus persisted in an isolated fashion over the occipital and postauricular scalp, corresponding to regions outside of the greater occipital nerve territory. On examination, these areas showed well-demarcated pink plaques with silvery scale, consistent with psoriasis. In contrast, the frontal, parietal, and vertex scalp regions were clear (Figure 2). She reported significant improvement in quality of life, reflected by her lack of use of topical medications even in areas with persistent rash.

**Figure 2:**
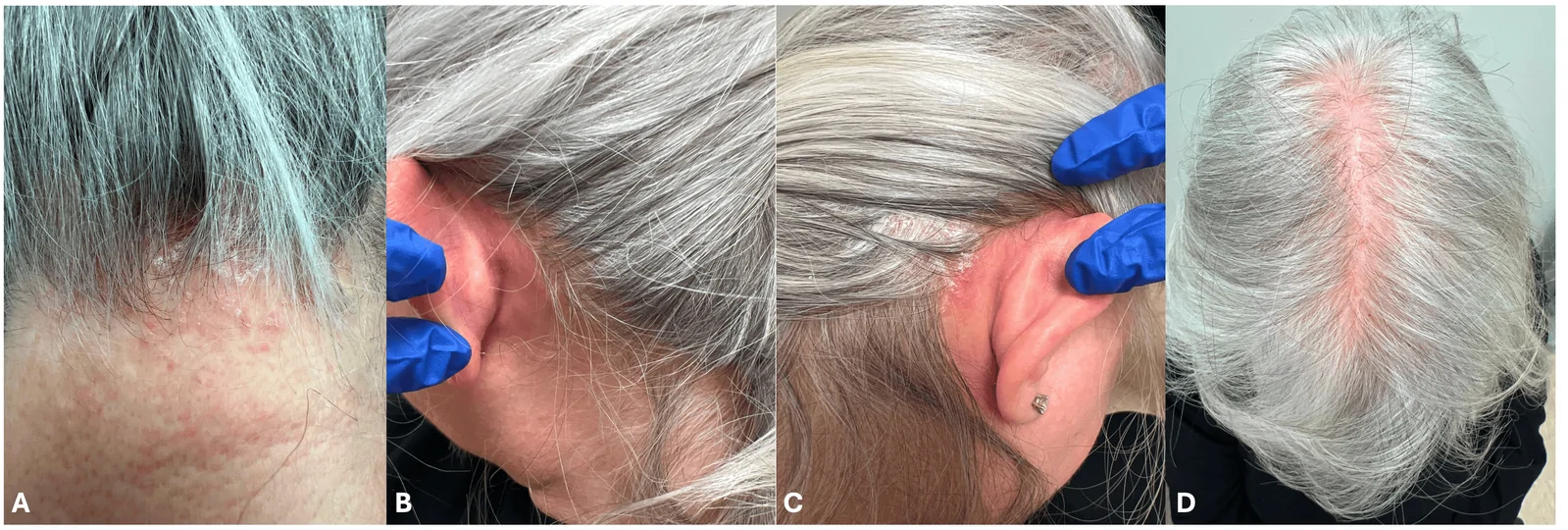
One hundred sixty-nine (169) days post-treatment, with notable improvement A). Occipital scalp B). Left postauricular scalp C). Right postauricular scalp D). Vertex scalp

## Discussion

This case highlights two notable features: the response to treatment was anatomically restricted and unexpectedly durable. Rather than generalized improvement across the entire scalp, rash clearance and itch resolution were confined to the territories corresponding to the bilateral greater occipital nerve distribution, whereas plaques in the adjacent occipital and postauricular scalp persisted. Given the sensory territories of the greater, lesser, and third occipital nerves overlap on the scalp, the zone of clearing should be interpreted as an approximation rather than an exact dermatomal map [6]. Nonetheless, the fact that improvement was isolated to the blocked nerve territory argues against spontaneous remission. 

Further, the duration of benefit in our case is also notable. Kambala et al. described 2 patients with neuropathic scalp pruritus who experienced 3 to 4 weeks of itch relief after bilateral greater occipital nerve blocks with 6 ml of 0.25% bupivacaine [10]. In contrast, our patient achieved 169 days of complete itch resolution with visible plaque clearance. As the Kambala cases involved neuropathic pruritus rather than psoriasis, our observation extends prior work by suggesting that occipital nerve blockade may have both antipruritic and local anti-inflammatory effects in scalp psoriasis [11]. Given that plain lidocaine provides only 30 to 60 minutes of anesthesia, the prolonged response further argues a mechanism beyond transient conduction blockade, potentially involving interruption of a feed-forward neuroimmune loop in which sensory nerve activation promotes inflammatory cytokine release that, in turn, further sensitizes cutaneous nerves, sustaining pruritus and plaque formation [12,13].

The proposed mechanism is supported by both clinical and experimental data. In a 2021 retrospective series and literature review, all 20 compiled cases with psoriasis and concurrent nerve injury demonstrated clearance or improvement of plaques within the affected neural territory, with only about one-third experiencing partial recurrence [14]. In a psoriasiform mouse model, cutaneous denervation reduced dendritic cell infiltration, T-cell number, and acanthosis [15]. The restoration of CGRP, but not substance P, reversed the improvement in acanthosis, supporting CGRP specifically as the key neuropeptide driving plaque formation [15]. Riol-Blanco et al. demonstrated that sensory nerves act upstream of the IL-23/IL-17 axis, as ablation of nociceptors prevented dendritic cells from producing IL-23 [16]. However, direct injection of IL-23 restored the inflammatory cascade even in denervated skin, suggesting that nerves drive the inflammatory process rather than being activated by it [16]. Within this axis, dendritic cell-derived (or exogenous, as above) IL-23 promotes T-cell production of IL-17, which acts on keratinocytes to drive proliferation, resulting in the prototypical scaling of psoriatic plaques. Directly relevant to our case, lidocaine has been shown to suppress abnormal nerve growth in psoriatic skin and block CGRP release from sensory neurons, thereby preventing CGRP from stimulating dendritic cells to produce IL-23 [13]. Consistent with these findings, an open-label study showed that of 32 patients with refractory, severe plaque psoriasis who completed a 1-week epidural lidocaine block, 87% achieved a Psoriasis Area Severity Index (PASI) score of 75, and 56% achieved a PASI score of 90 by 48 weeks [9]. We therefore hypothesize that peripheral nerve blockade may transiently reduce sensory nerve signaling and CGRP release within the innervated territory, decreasing dendritic cell IL-23 production and downstream IL-17-mediated plaque maintenance; ultimately leading to spatially confined yet durable psoriatic improvement. Together, these studies provide biological plausibility that the anatomically confined and prolonged response observed in our case reflects an interruption of a self-amplifying CGRP/IL23/IL17 neuroimmune circuit.

It is important to note that this case report does have multiple limitations. First, we are unable to establish causality from a single observation. Additionally, the underlying mechanistic pathophysiology regarding a feed-forward neuroimmune circuit is simply a hypothesis, which, to our knowledge, has not been rigorously scientifically validated. We have also considered that the clinical response may represent the natural progression of disease, including spontaneous remission or delayed effects of prior treatments. There is also the placebo effect after procedural intervention, which could be contributing. The anatomic specificity of improvement in this patient may argue against these alternative explanations, but they cannot be completely excluded. 

## Conclusions

In conclusion, the patient experienced both complete resolution of pruritus and visible plaque clearance isolated to the bilateral greater occipital nerve territory for more than five months after a single lidocaine injection, without the use of other systemic or topical therapies and with noted disease in the adjacent scalp. Injectable lidocaine is an inexpensive resource found in most dermatology offices, given its use in multiple clinical scenarios. Thus, this may represent a simple and rapid in-office procedure with durable results.
